# The Reproductive Journey in the Genomic Era: From Preconception to Childhood

**DOI:** 10.3390/genes11121521

**Published:** 2020-12-19

**Authors:** Sandra Garcia-Herrero, Blanca Simon, Javier Garcia-Planells

**Affiliations:** IGENOMIX, Valencia, 46980 Paterna, Spain; sandra.garcia@igenomix.com (S.G.-H.); blanca.simon@igenomix.com (B.S.)

**Keywords:** genetic testing, reproductive health, next-generation sequencing, whole exome sequencing, perinatal care

## Abstract

It is estimated that around 10–15% of the population have problems achieving a pregnancy. Assisted reproduction techniques implemented and enforced by personalized genomic medicine have paved the way for millions of infertile patients to become parents. Nevertheless, having a baby is just the first challenge to overcome in the reproductive journey, the most important is to obtain a healthy baby free of any genetic condition that can be prevented. Prevention of congenital anomalies throughout the lifespan of the patient must be a global health priority. Congenital disorders can be defined as structural or functional anomalies that occur during intrauterine life and can be identified prenatally, at birth, or sometimes may only be detected later during childhood. It is considered a frequent group of disorders, affecting 3–6% of the population, and one of the leading causes of morbidity and mortality. Congenital anomalies can represent up to 30–50% of infant mortality in developed countries. Genetics plays a substantial role in the pathogenesis of congenital anomalies. This becomes especially important in some ethnic communities or populations where the incidence and levels of consanguinity are higher. The impact of genetic disorders during childhood is high, representing 20–30% of all infant deaths and 11.1% of pediatric hospital admissions. With these data, obtaining a precise genetic diagnosis is one of the main aspects of a preventive medicine approach in developed countries. The field of reproductive health has changed dramatically from traditional non-molecular visual microscope-based techniques (i.e., fluorescence in situ hybridization (FISH) or G-banding karyotype), to the latest molecular high-throughput techniques such as next-generation sequencing (NGS). Genome-wide technologies are applied along the different stages of the reproductive health lifecycle from preconception carrier screening and pre-implantation genetic testing, to prenatal and postnatal testing. The aim of this paper is to assess the new horizon opened by technologies such as next-generation sequencing (NGS), in new strategies, as a genomic precision diagnostic tool to understand the mechanisms underlying genetic conditions during the “reproductive journey”.

## 1. Introduction

The odyssey of an infertile couple to become parents can be compared to the odyssey of those parents who realize that their child has some type of problem or illness but there are no answers or name for that “condition“. It can also be compared to adults that suffer from a genetic condition and want to know what it is, how to deal with it and their reproductive options, embarking on a long, emotionally draining and financially costly journey.

There is an indisputable advancement that the emergence of molecular genetic techniques, such as next-generation sequencing (NGS), has allowed, which is to standardize pathways to achieve an accurate diagnosis. Genomic high-resolution and high-throughput technologies have opened new diagnostic pathways, allowing a more personalized clinical management paired with new challenges, such as the use, management and interpretation of generated databases needing great bioinformatic support.

## 2. The Reproductive Journey

### 2.1. First Stage: Pre-Conceptional Care

**Figure 1:**
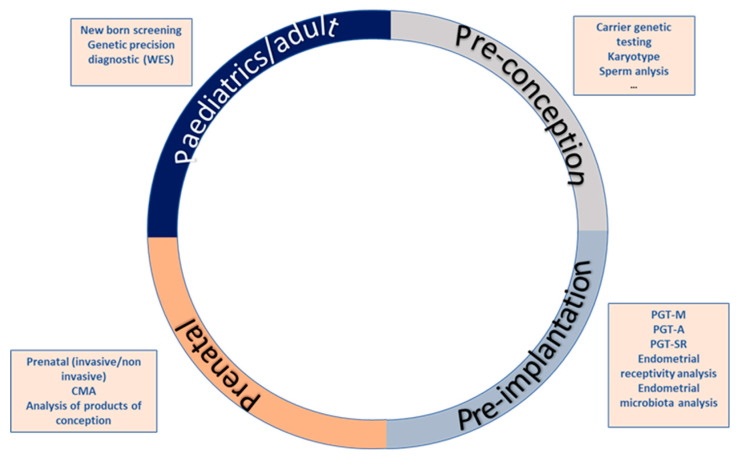

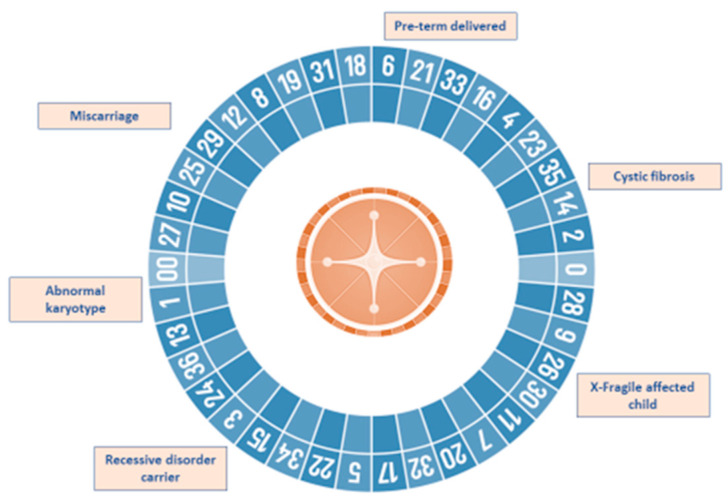
Reproductive journey vs. reproductive roulette: Genetic analysis can be implemented at any stage of the reproductive journey, starting from preconception to detect genetic carriers of frequent diseases, pre-implantation to ensure chromosomal and genetically normal embryos, prenatal diagnosis and lastly, for newborn screening of common and actionable diseases. The reproductive roulette is a term that aims to explain the unknown risk of having any form of genetic disease given the risk factors of the parents or purely by chance. The possibility of reducing this probability can be done by an adequate and directed genetic analysis or screening approaches.

Increasingly often, more couples assess their reproductive potential without acknowledging the reproductive roulette of the risk for an associated genetic disease (Figure 1). The growing knowledge on the impact of genetic diseases in soon to become newborns, as well as the development of new technologies, has led to an increase of the pre-conceptional care field. Nevertheless, genetics is not the only area covered by pre-conceptional assessment. Genetic analysis can be implemented at any stage of the reproductive journey, starting from preconception to detect genetic carriers of frequent diseases like cystic fibrosis, hemophilia or fragile X syndrome, pre-implantation to ensure a chromosomal and genetically normal embryo is transferred, decreasing the risk of monogenetic disease like Duchenne muscular dystrophy, aneuploidies such as Down’s syndrome or structural diseases like DiGeorge’s syndrome or Prader Willi syndrome. Genetic analysis is also useful for prenatal diagnosis of these kinds of diseases, high-risk pregnancies and in case of spontaneous abortions, the analysis of the products of conception. Lastly, it can be utilized to perform newborn screening of common and actionable diseases, personalized genetic analyses such as single gene analysis for monogenic diseases and genetic panels or whole exome sequencing for complex or clinically unspecific diseases (Figure 1).

The WHO (World Health Organization) defines preconception care as the provision of biomedical, behavioral and social health interventions for women and couples before conception occurs. Its main aim is to improve maternal and child health, in both the short and long term [1]. Preconception counseling must cover all known barriers that may have a detrimental effect on fertility or pregnancy which include:

- An evaluation of the overall well-being
- Medical history
- Surgical history
- Social and behavioral history
- Medication
- Occupational and education risks

There are many areas addressed by preconception care assessment including nutrition, environmental conditions, toxic habits (i.e., tobacco and alcohol consumption), mental health and genetic conditions. We are going to focus on the last one, genetic conditions.

Most genetic disorders that result in sterility or childhood death are caused by recessive mutations. Nonetheless, these variants can cause devastating diseases like cystic fibrosis when the patient carries both copies of the mutation. It is estimated that humans carry an average of one to two mutations per person that can cause severe genetic disorders or prenatal death when two copies of the same mutation are inherited [2]. This means that if two carriers of the same mutation have a child, it could be affected by a genetic disease.

Currently, there are many genetic tests that assess the “mutational state” of a patient or a couple to reduce the probability of having a baby with a genetic disorder. Genetic carriers screening based on NGS test the existence of mutations causing a vast number of recessive genetic conditions in an individual, that can be passed on to their offspring if the couple carries the mutation. Even though the standard of practice is to offer carrier testing only to those individuals who have a strong family history of a genetic disorder, or a history of genetic disorder in the partner and/or relatives of identified carriers, only a minority of carrier couples are identified. These reduced indications for testing can lead to children affected by recessive disorders with no known family or medical history [3].

Autosomal/X-linked recessive disorders are more frequent than autosomal/X-linked dominant because the latter present a higher deleterious effect. The reproductive approach is different in these cases because patients are not only carriers, but they also suffer the illness, and so are aware that they can transmit the genetic condition to 50% of their offspring.

When it is known that an autosomal and/or dominant disease is present in a couple, preconception counselling is crucial. During this process, we must evaluate the medical and family history to obtain an accurate clinical diagnosis. From here, the next step will be to carry out the most appropriate genetic analyses to identify the molecular cause of the disease, an essential requirement for any subsequent family study, including prenatal and pre-implantation analyses.

### 2.2. Second Stage: Pre-Implantation Diagnosis

At this stage of the reproductive journey, it is useful to group together couples with known reproductive problems (infertility, miscarriages, previously affected child…) as well as those couples that have never tried but know there is a genetic condition running in the family. Therefore, all of them could benefit from assisted reproduction techniques such as pre-implantation genetic testing for monogenic diseases (PGT-M) and pre-implantation genetic testing for aneuploidies (PGT-A).

PGT-M allows us to detect embryos affected by a known monogenic that has been previously detected in their parents. Molecular technologies used to perform the PGT-M test were several, for example:

- Multiplex PCR (Polymerase Chain Reaction): Multiplex PCR uses targeted primers designed specifically for the mutation of interest combined with other markers for linked short tandem repeat (STR) markers.
- Whole-genome amplification (WGA).
- Karyomapping: High-density SNP (Single Nucleotide Polymorphism) array that allows evaluation of DNA haplotypes).
- Sanger sequencing.
- Multiplex Ligation-dependent Probe Amplification (MLPA) [4].

Besides genetic disorders caused by gene mutations/variations, other genetic conditions can have an impact on fertility, pregnancy, parents and newborns: the so-called chromosomal disorders.

As women age, their fertility declines and there is an increased risk of numerical and structural chromosomal abnormalities, which can lead to implantation failure, early pregnancy loss, greater risk of congenital birth defects or severe chromosomal congenital diseases such as Down’s and Patau syndromes. Aneuploidy ranks as the most common genetic abnormality accounting for approximately 50% of miscarriages. More than half of the embryos produced by in vitro fertilization (IVF) are aneuploid [5].

The process of detecting numeric or structural chromosomal abnormalities for the purpose of embryo selection is generally referred to as pre-implantation genetic testing for aneuploidies (PGT-A), introduced in the 2000s to increase implantation and pregnancy rates, decrease miscarriage rates and the risk of aneuploid offspring, as well as decrease the time to conceive [4,6,7]. Early PGT-A utilized fluorescence in situ hybridization (FISH) screening. However, data from several studies questioned the efficiency of FISH screening [8,9,10,11], which is restricted due to the limited panel of chromosomes that it is able to analyze. In recent years, PGT-A using FISH screening has been initially replaced by comprehensive approaches, including comparative genomic hybridization arrays (CGH) or single nucleotide polymorphism microarrays, and more recently, by next-generation sequencing (NGS)-based techniques.

Currently, embryo biopsy is required for PGT-A testing. In the event of a poor blastocyst quality at biopsy, new effective approaches involving the sequencing of cfDNA (cell free DNA) secreted into the culture medium from the human blastocyst have been developed. In addition, PGT-A could mitigate the potential adverse effects associated with embryo biopsy [12,13].

Aside from the assessment of the embryo´s mutational/chromosomal status, additional genetic tests assessing fertility, based on high-throughput techniques such as NGS, are beyond the scope of an ordinary clinical practice to increase the reproductive chances of a couple, i.e., endometrial receptivity analysis and more recently, endometrial microbiome test [14,15].

### 2.3. Third Stage: Prenatal Diagnostis

Prenatal screening is the risk estimation of fetal aneuploidies based on high-resolution ultrasound scans, in order to assess ultrasonographic markers including nuchal translucency, combined with biochemical determinations in maternal blood samples of free beta-subunit of human chorionic gonadotropin (fβ-hCG) and pregnancy-associated plasma protein-A (PAPP-A) in the first trimester, and the alpha-fetoprotein (AFP) and beta-human chorionic gonadotropin (βhCG) in the second trimester [15]. If this risk of congenital defect is high, invasive procedures such as chorionic villus sampling (CVS) or amniocentesis are recommended [16]. Fetal chromosomal assessment traditionally performed using Giemsa banding (G-banding) on cultured cells in metaphase is considered as the gold standard detection method [17,18]. Although the accuracy and reliability of this technique is very high, 99.4–99.8% and 97.5–99.6% for amniocentesis and CV respectively [19], there are considerable disadvantages that must be highlighted: prenatal tissue must be cultured for several days to obtain metaphase nuclei prior to analysis, increasing maternal anxiety and the risk of fetal loss up to 0.5–2% due to an invasive technique used for fetal tissue extraction (i.e., amniocentesis to obtain amniotic fluid).

Currently, a rapid noninvasive prenatal test for the most common aneuploidies in the live newborn (i.e., Down or Turner syndrome) can be performed by sequencing fetal DNA present in maternal blood. The genomics-based non-invasive prenatal test (NIPT) could be considered as a candidate to replace the conventional karyotype as a first-tier test in unselected populations of pregnant women undergoing aneuploidy screening or as a second-tier test in pregnant women considered to be high risk after first-tier screening for common fetal aneuploidies [20].

Despite that cytogenetic conventional karyotype has been considered the gold standard for chromosomal assessment, new molecular microarray-based genomic copy-number techniques like chromosomal microarray (CMA) present some advantages.

The resolution of chromosomal analysis by karyotyping is limited to 5–10 Mb in size [21]. Most chromosomal anomalies identified in early pregnancy are aneuploidies, which are detected using conventional karyotyping. CMA resolution is higher than karyotype, therefore offering additional diagnostic benefits by revealing sub-microscopic imbalances or copy-number variations (CNV) that are too small to be detected using a standard G-banded chromosome preparation. Clinically significant copy-number variations not identifiable by standard karyotyping occur in 1–1.7% of routine pregnancies [22]. Most of these CNVs are responsible for:

- A variety of phenotypes.
- Multiple malformations.
- Congenital anomalies.
- Intellectual disabilities.
- Developmental delay.
- Epilepsy.
- Cerebral palsy.
- Neuropsychiatric disorders [23].

Alternatively, there are trade-offs, however, DNA extraction needed for prenatal purposes in order to perform a CMA still requires invasive techniques such as amniocentesis or chorionic villus sampling. Furthermore, a high resolution increases the probability of incidental findings of unknown clinical significance that, in turn, add a level of complexity to the genetic counselling as well as parent anxiety. We must take into consideration that CMA does not detect polyploidies or balanced rearrangements. In the vast majority of cases, the presence of a balanced rearrangement does not imply major clinical significance for the ongoing pregnancies, but there are still reproductive ramifications for future pregnancies if one of the parents is a carrier [24].

Although aneuploidies are the most frequent genetic alteration during the prenatal stage, as well as one of the main genetic causes of congenital defects (10–15%), monogenic alterations are of considerable importance throughout this stage, reaching up to 10% of the congenital defects.

Some of the clinical features of these monogenic disorders, especially those associated with syndromic forms, can be identified throughout pregnancy by ultrasonography analyses. In these cases, and depending on the clinical impression, specific tests can be used to analyze certain genes or variants, as well as more complex and nonspecific technologies, such as CMA, NGS gene panels or whole exome sequencing (WES), when a precise clinical guidance is not possible. In any case, when an ultrasound finding is detected during pregnancy, that pregnancy is labelled as high risk for a genetic disease, therefore an invasive test will be indicated to obtain a fetal sample to analyze using the most suitable technique depending on the type of ultrasound finding.

Another significant and relatively frequent issue is when the couple first finds out about the presence of a genetic disease or knows that they are at high risk of being a carrier during an advanced stage of their pregnancy. As we have described previously, it is strongly recommended to face this situation in the pre-conceptional stage in order to approach the diagnostic process with sufficient guarantees and time. Once the pregnancy has started, the gestational age and prenatal diagnosis can be time-limiting, being critical in some complex cases. In any case, and whenever possible, it is recommended to identify the molecular cause of the familial disease prior to taking the fetal sample using an invasive approach.

Unfortunately, miscarriages are the most common complication during early pregnancy. Clinically recognized pregnancy losses occur in approximately 15–25% of pregnancies, most of them occurring during the first trimester [25]. Although there are many known causes and risk factors for early pregnancy loss, about 60% of those cases are caused by sporadic chromosomal abnormalities which are usually numerical (86%) [26,27,28,29]. These cytogenetic anomalies include autosomal trisomies (27%), polyploidies (10%), chromosome X monosomy (9%) and structural rearrangements (2%) [30]; double trisomies, as well as multiple trisomies, which are infrequent, have an incidence of about 0.7% [31,32].

Until now, products of conception (POC) studies have been carried out using cell culture followed by conventional karyotyping. However, when using these techniques, the incidence of chromosomal abnormalities in miscarriages in the general population ranges between 40% and 80%, depending on the culture methods adopted [33,34]. Proper chromosomal analysis of POC samples is not always feasible, for several reasons:

- Cell culture growth failure (the failure rate in POC samples cultured after curettage ranges between 5% and 42% [35]).
- Suboptimal chromosome preparations.
- Maternal cell contamination (MCC).
- Low-resolution limit that does not allow the detection of submicroscopic deletions and duplications that can cause miscarriages.

Molecular biology techniques such as NGS or CMA that are culture-independent can avoid such limitations, increasing the karyotype resolution [36,37]. Given this, new genomic technologies are positioning themselves as the first-choice technologies for the analysis of miscarriages and POC.

Whole exome sequencing (WES) is very useful for the detection of alterations in the sequence of any gene that may be related to the potential genetic condition that may have caused the spontaneous termination of the pregnancy in progress. This is especially important in the second trimester of gestation when monogenic disorders acquire a higher frequency. In these cases, identification of the molecular causes of the miscarriage can be very useful to prevent new similar situations in the couple.

When miscarriage occurs in an advanced pregnancy, the clinical and anatomopathological evaluation of the fetus can be very useful to guide the genomic analysis. When clinical assessment is not possible, WES provides a high capability to identify sequence variants in genes associated to complex syndromes, but also, the optimization of bioinformatic analyses, making possible the identification of copy-number variations in these cases.

### 2.4. Fourth Step: Newborn Screening and Neonatal Care

Currently, 3% of live newborns will have a congenital alteration despite great efforts made in the different stages of the reproductive process, growing capacities of available technologies and the implementation of prevention programs. This is because, on one hand, technologies, although increasingly precise, are not infallible, and on the other, the use of prevention techniques and programs are not universal.

In this stage, as in other previous stages, we will be able to apply screening measures in order to reduce the impact of congenital diseases. An example would be extended neonatal screening aimed for early identification of apparently healthy newborns that are at immediate risk of a congenital disease if an early and accurate diagnosis is not established and therapeutic measures are not taken as soon as possible.

The neonatal screening allows us to detect a wide number of genetic disorders, causing health problems starting in infancy or early childhood, mainly metabolic disorders like phenylketonuria. Newborn screening programs are well-established as the standard of care in most developed countries, but the number of diseases and approaches differ between countries and health systems. Early detection and treatment can help prevent inborn errors of metabolism, intellectual and physical disabilities and life-threatening illnesses during the first hours of life. The advent of next-generation sequencing has resulted in attempts to expand the use of DNA sequencing in newborn screening to improve diagnostic and prognostic utility. Currently, in the market, we can find different commercial options for newborn screening panels that can detect, not only the most common metabolic disorders, but a great number of genetic disorders, or even gene susceptibility. Still, unexpected and medically irrelevant incidental findings must be carefully considered [38].

On the other hand, we can also apply diagnostic methods in those neonates who have developed symptoms, especially for newborns admitted to the intensive care unit when disease progression is extremely rapid and a quick molecular diagnostic is relevant for clinical decision making, establishing a prognosis, defining specific therapeutic measures and providing genetic counselling and access to family studies aiming to reduce the risks of recurrence in the family. Monogenic diseases have a high impact in the neonatal morbimortality, accounting for ~20% of infant deaths and ~18% of pediatric hospitalizations. Genomic testing of these patients aims to provide a comprehensive molecular diagnosis that allows for early intervention of the patient and proper genetic counseling of the family in order to reduce the time spent in the diagnostic odyssey [39,40]. These tests provide a high clinical utility and are cost-effective, especially in patients involved in neonatal intensive care units.

Both genetic assessment and diagnosis have a special impact during this stage of the reproductive journey, especially in young adults that may be developing a career, forming partnerships and potentially becoming parents. Pre-symptomatic testing may affect many facets of their future lives as well as the future of their upcoming families [41], but also raises profound ethical challenges.

NGS technologies, especially introduction of the WES, has become a turning point, especially in the rare genetic diseases research. It has allowed development and implementation of strategies to uncover the mechanisms behind all rare diseases to sketch a “molecular atlas” showing links between molecular genetic profiles and states of health or disease. Rare genetic conditions affect around 2–3% of the worldwide population, usually causing diseases that drastically reduce life expectancy and quality of life as well as reproductive consequences in their offspring.

## 3. Genomic Precision Diagnostic

Recent advent of genomic technologies and its clinical applications has allowed the scientific community, especially those involved in patient care and clinical management, to evolve from a diagnosis-based approach of fragmented or isolated data to a diagnostic-based approach of the “overall picture” point of view. This can be done as a result of huge datasets, using their unique genetic and physiologic characteristics to tailor the diagnosis and treatment of individual patients.

To reach an accurate and reliable genetic diagnosis, or in other words, to interpret these data in a meaningful and useful manner for the patient’s clinical management, it is mandatory to develop bioinformatic tools, including novel data pipelines or computational tools, and also, acquire knowledge to manage and to interpret combined analysis of multi-dimensional data produced by these technological and scientific innovations. This is where clinical bioinformatics comes into play as an essential element to integrate laboratory and clinical data as well as the use of database-computational methods, algorithms, customized pipelines and other resources and methods, including machine learning or even artificial intelligence [42].

Whole exome sequencing (WES) and whole genome sequencing (WGS) are the most recent technologies based on NGS that are developed for genomic diagnostic purposes when a genetic disorder for which single-gene or limited gene testing fails to provide a genetic explanation. WGS and WES—wherein the genome or the protein-coding parts of the genome are sequenced in its entirety—would be an option to replace panel tests in the near future. There is no doubt that genomic tools, especially WES and WGS, give us a global view of the disease, increasing the knowledge on the underlying mechanism causing this rare genetic condition, but also increases the probability to find variants associated with other health conditions which may or may not be medically actionable and are unexpectedly so named secondary or incidental findings. How to report and manage this secondary finding as well as how to assess patients are some of the main genomic diagnostic challenges [43].

## 4. Conclusions

It is estimated that 350 million people globally suffer from a genetic disorder causing a rare condition. Genetic disorders can be assessed in several stages of our life, generating a different impact in our lifestyle and personal decision-making, in particular those involved with our reproductive options.

Frequently, searching for an answer and an accurate diagnosis in those patients suffering from a genetic disorder can turn into an odyssey, especially in those cases where children or pregnant women are involved.

**Figure 2:**
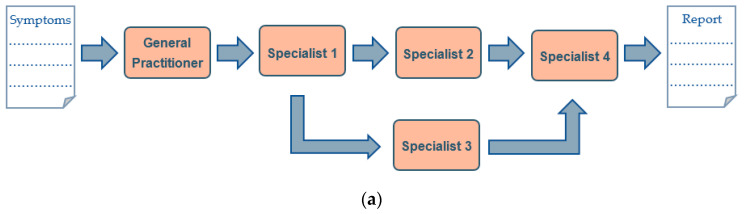

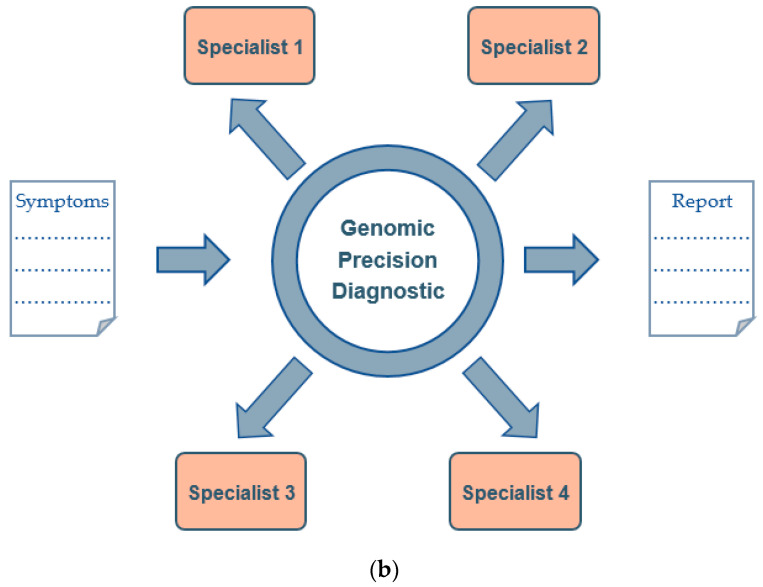
Classic genetic diagnostic (**a**) vs. Genomic diagnostic (**b**) flow-charts: Genomic Precision Diagnostic is an innovative approach that ultimately not only provides a precise diagnosis of a genetic alteration at every stage of the reproductive journey, it also alleviates the healthcare burden of a patient that has to go from specialist to specialist, increasing the degree of uncertainty and preoccupation. This can decrease the economic cost, psychological deterioration of the patient and the healthcare provider, and all in all, simplify the diagnosis for a more directed treatment and patient care, allowing the patient to be the center of the diagnostic axis.

Sometimes, patients and their families bounce back and forth from physician to specialist and back again only to receive multiple misdiagnoses. Genetic diagnosis serves to provide a linear or step-by-step flowchart with plenty of medical tests, reports and documentation of clinical manifestations. These lead to the concatenation of several visits, usually to different specialists, only to end up with the final step, which was a genetic analysis, usually by Sanger sequencing or NGS of a limited/selected gene panel when there was a suspicion of a genetic disorder. The emergence of a massive gene analysis tool like NGS has changed this diagnostic workflow in genetic diseases by providing a higher diagnostic yield, rapid, powerful and cost-effectiveness alternative for genetic analysis in the early stages of the process (Figure 2). This new workflow has drastically reduced waiting times and anxiety that many patients have to undergo to know their prognosis [44].

Currently, NGS-based tools can point to the implication of a single gene (or a small number of genes) and help establish a rapid diagnosis in just a few weeks or even less in a large percentage of cases.

Lately, we have experienced a noticeable increase in the demand of NGS technologies in the clinical setting. Increasing the diagnostic precision of these technologies has been one of the main levers for change, alongside the optimization of processes, reduction of costs and development of new applications. These adjustments contribute to an improvement in the diagnostic capacity, improving the life of patients with monogenic and widely known diseases, patients with heterogeneous conditions, gene-associated phenotypes or even complex differential diagnoses.

**Figure 3:**
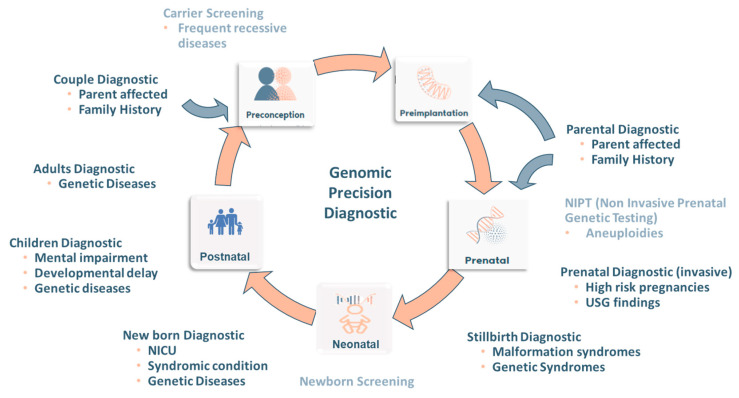
Closing the circle of the Reproductive Journey: Impact of the genomic diagnostic (dark blue) and screening (light blue) application along the different stages of the reproductive journey. Blue-gray arrows show new “access ways” to the reproductive journey driven by a precise diagnostic.

Genomics provides the tools and knowledge necessary to achieve an accurate diagnosis, making a difference in the lives of a growing number of patients suffering from genetic diseases. Relying on an accurate diagnosis enables a greater availability to different reproductive options, allowing the “reproductive journey” to be a comfortable and safe experience for the patient (Figure 3).
